# 2414. Pulling the Cord: Removal of Urinary Catheters before Urine Culture in a Cardiothoracic-Surgery ICU

**DOI:** 10.1093/ofid/ofad500.2034

**Published:** 2023-11-27

**Authors:** Michael Ben-Aderet, Meghan Madhusudhan, Jonathan Grein, Carissa Drucker, Michael Nurok, Alice Chan, Sowmya Kalava, Moises R Vargas, Jake Cornett, Jill Yaung, Katrina Harper-Kirksey, Eliza Granflor

**Affiliations:** Cedars Sinai Medical Center, Los Angeles, CA; Cedars-Sinai Medical Center, Los Angeles, CA; Cedars-Sinai Medical Center, Los Angeles, CA; Cedars-Sinai Medical Center, Los Angeles, CA; Cedars-Sinai Medical Center, Los Angeles, CA; Cedars Sinai, Walnut, California; University of California Los Angeles, Los Angeles, California; University of California Los Angeles, Los Angeles, California; Cedars-Sinai Medical Center, Los Angeles, CA; Cedars-Sinai Medical Center, Los Angeles, CA; Cedars Sinai Medical Center, Los Angeles, CA; Cedars-Sinai Medical Center, Los Angeles, CA

## Abstract

**Background:**

Detection of asymptomatic bacteriuria (ABU) can lead to significant consequences, including over-prescription of antibiotics. Numerous studies have demonstrated that urinary catheters (UCs) are quickly colonized with bacteria, even when UTI is not present, and obtaining cultures from catheters leads to increased detection of ABU and increased Catheter Associated Urinary Tract Infection (CAUTI) rates. In our Cardiac Surgery ICU (CTICU), where UC utilization is high with limited opportunities for removal, we implemented an ABU reduction intervention consisting of replacing UCs prior to urine cultures.

**Methods:**

In July 2021, we implemented a new urine culture stewardship process in the CTICU. When a patient with a UC required a urine culture, a urinalysis (UA) was ordered. If pyuria was present, the UC would be replaced, and a new urine culture (direct culture or a UA with reflex to culture) would be drawn from the new UC. This process was implemented as a recommendation, and compliance was encouraged by physician and nursing leadership, but not prospectively monitored. Retrospective review was done comparing urine culture positivity, antibiotic use and CAUTI rates in the period after the intervention with 2 full calendar years prior to the intervention. We also looked at catheterization rates and urology consultation rates (as a marker for urinary tract injury)

**Results:**

Post-intervention, we saw a 43% reduction in the rate of urine cultures (4.9 to 2.8 cultures / 1000 patient days, p-value < 0.001), a 67% reduction in rate of positive urine cultures (1.2 to 0.4 positive cultures / 1000 patient days, p-value < 0.001), and an 87.5% reduction in CAUTI rate (1.6 to 0.2 CAUTIs / 1000 catheter days, p-value < 0.001). There was no significant change in urinary catheterization rate, gram-negative antibiotic use, or rate of urology consultation.

**Figure 1:**
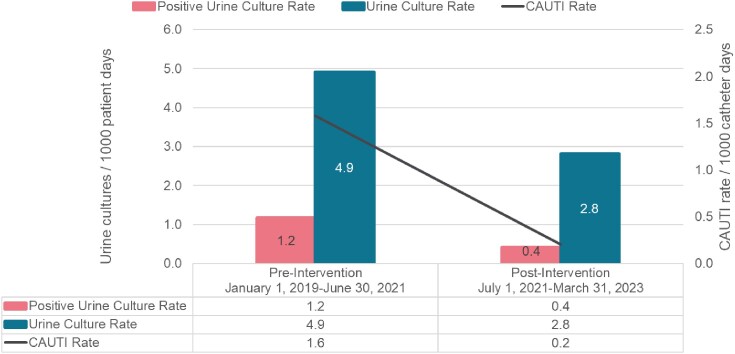
Urine culture, positive urine culture and CAUTI rate before and after the intervention

**Figure 2:**
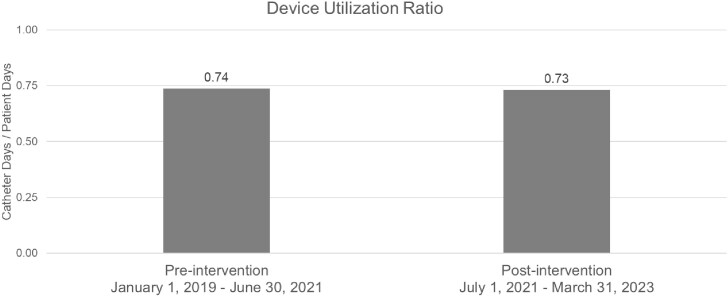
Urinary catheter utilization ratio before and after the intervention.

**Figure 3:**
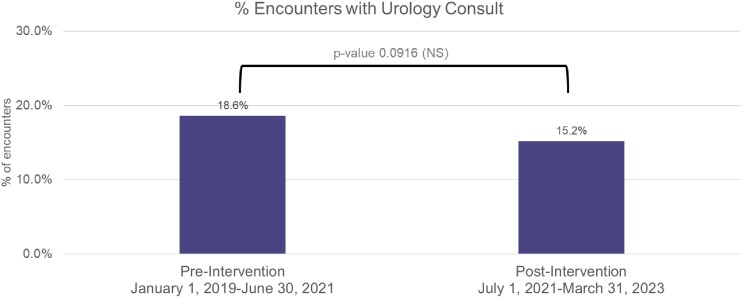
Proportion of encounters with a urology consult before and after the intervention

**Conclusion:**

We observed a dramatic reduction in CAUTI, positive urine cultures and all urine cultures with no increase in Urology consultations or change in UC utilization. While the reduction in reportable CAUTI is important and highly relevant, further work is needed to explore the full potential for this intervention regarding antimicrobial stewardship.

**Disclosures:**

**All Authors**: No reported disclosures

